# Methodological considerations for determining the volume and intensity of drop jump training. A systematic, critical and prepositive review

**DOI:** 10.3389/fphys.2023.1181781

**Published:** 2023-04-21

**Authors:** Raynier Montoro-Bombú, Hugo Sarmento, Carlo Buzzichelli, Nelio Alfano Moura, Juan José Gonzáles Badillo, Amândio Santos, Luís Rama

**Affiliations:** ^1^ University of Coimbra, Research Unit for Sport and Physical Activity (CIDAF), Faculty of Sport Sciences and Physical Education, Coimbra, Portugal; ^2^ Faculty of Exercise and Sport Science, University of Milano, Milan, Italy; ^3^ Chinese Athletics Association, Beijing, China; ^4^ Faculty of Sport, Pablo de Olavide University, Seville, Spain

**Keywords:** drop jump, depth jump, jumping program, plyometric training, plyometrics

## Abstract

This systematic review provides critical and propositional information on criteria for determining the volume and intensity of drop jumps during plyometric training programs. Eligibility criteria were defined according to PICOS: Participants: male or female athletes, trained or recreationally active (16–40 years). Intervention duration: more than 4 weeks. Comparators: passive or active control group during a plyometric training program. Outcomes: information on improvement with Drop Jump or Depth Jump, with other jumps, acceleration, sprint, strength, and power output. Design: randomized controlled trials. We searched articles published in PubMed, SPORTDiscus, Web of Science, and Scopus. The search was conducted until 10 September 2022 for English-language articles only. The risk of bias was assessed using Grading of Recommendations, Assessment, Development and Evaluation (GRADE) for randomized controlled studies. We identified 31495 studies, of which only 22 were included. We found that six groups presented results with women, 15 presented results with men, and the remaining four presented mixed studies. Of the 686 people recruited, 329 participants aged 25.79 ± 4.76 years were involved in training. Methodological problems in training intensity, volume distribution, and individualization were noted, but methodological recommendations for their solution are also provided. It is concluded that the drop height should not be understood as the intensity determinant of plyometric training. Intensity is determined by ground reaction forces, power output, and jump height, among other factors. Furthermore, the athletes’ experience level selection should be based on the formulas recommended within this research. These results could be helpful for those who intend to conduct new plyometric training programs and research.

## Introduction

Plyometric exercises have been used for research and training programs since the 1960s. This training method has grown in popularity over the last 12 years. A PubMed search for the term “plyometric” yields 237 research articles published between 1983 and 2010, while 301 were published between 2021 and 2022 alone, highlighting the importance of the topic.

Among the most classic exercises are the Depth Jump (DeJ) and the Drop Jump (DJ), where the athlete stands upright on a box, and keeps his hands on his hips, determines the starting leg, and then performs a free fall in order to provoke a high force against the ground and take off in the shortest possible time in the case of the DJ, but reach a greater jump height (JH) in the case of the DeJ (Clutch et al., 1983) The differences between these tasks are presented in appendix 1. During the free fall, the neuromuscular system prepares to counteract the effects of gravity, a phase in which neuromuscular pre-activation (Taube et al., 2012; Di Giminiani et al., 2020) and proprioceptive receptors which are integrated into the central nervous system (Taube et al., 2012) intervene to ensure an effective landing. The athlete then comes into contact with the ground, where the stretch-shortening cycle (SSC) takes place (Dietz et al., 1979; Schmidtbleicher and Haralambie, 1981; Horita et al., 2003; Turner and Jeffreys, 2010; Pedley et al., 2022). This consists of an eccentric phase where the muscle-tendon unit of the already pre-activated contractile component (Bosco et al., 1982b; Taube et al., 2012) starts a braking phase and lengthens, causing an accumulation of elastic energy at the tendons (Ishikawa et al., 2006) and the production of mechanical impulse. This is followed by stabilization time (Wikstrom et al., 2004; Flanagan et al., 2008) and a transition from eccentric to concentric, where velocity = 0 is reached, after which the concentric phase of the movement begins, where the elastic energy is released as a result of the shortening of the muscle-tendon unit (Ishikawa et al., 2006), summating to the myotatic stretch reflex for a more powerful concentric action (Dietz et al., 1979).

The science fails to identify the first to introduce the term “plyometric,” and it is shared between Margaria. R and Wilt. F (Wilt, 1976), The term plyometric is thought to be derived from the Greek “plio,” which is associated with longer or wider and, “metric,” which means to measure. As far as we reviewed, the earliest research reported in the scientific literature on plyometric exercise is provided by Asmussen and Bonde-Petersen (1974) and by Verkhoshansky and Chernousov (1974). Asmussen and Bonde-Petersen (1974) found that there was a 5% improvement in JH comparing the Squat Jump (SJ) to the countermovement Jump (CMJ) and 11% comparing the SJ with the presently known 40 cm DJ, demonstrating that a negative work phase preceding the jump, significantly increases jump height up to a point, as previously thought. Y. Verkhoshansky himself (Verkhoshansky, 2006) assumes that this concept of plyometric work had previously been characterized by Sechenov and Mikhailovich (1863), when he described the spring function of the muscle. The former, in his work with jumping athletes, exploited the kinetic energy accumulated in the legs as a result of free falls and emphasized that the plyometric method is not a simple deformation of the muscle as a result of stretching, but that this stretching has to be fast (Verkhoshansky, 2006). According to this criterion, he proposed a range of fall heights (FH) for the training of the lower limbs (0.10–2.2 m), and the concept of “optimal plyometric load” (0.75 m) based on the average power output, which has been strongly criticized to this day (Bobbert et al., 1987a; Bobbert et al., 1987b; Baechle et al., 2008). During the period 1976–1982, considerable contributions were made by C. Bosco, who, for instance, found that athletes improved their CMJ by 11% and their DJ by 15% by using DJ in their training program (Komi and Bosco, 1978b; Bosco and Komi, 1979a; 1980; Bosco and Komi, 1979b; Bosco et al., 1981; Bosco et al., 1982a; Bosco et al., 1982b; Bosco et al., 1982c). Bosco also compared the jumping ability of men and women (Komi and Bosco, 1978a). Together with Verkhoshansky’s, Bosco’s work on plyometric and ballistic exercises is considered seminal when it comes to of plyometric training (Bosco et al., 1982c; Bosco et al., 1982d; Viitasalo and Bosco, 1982; Bosco et al., 1983a; Bosco et al., 1983b; Bosco et al., 1986), nevertheless, plyometric training has continued to evolve over the years (Baechle et al., 2008; Morin et al., 2019).

During that time, other researchers also made contributions to the theory of plyometric training. Cavagna (Margaria et al., 1960; Cavagna et al., 1965; Cavagna and Citterio, 1974) was among the first to provide an experimental justification for plyometric training, conducting experiments on the negative phase of jumping contributing to the action of the contractile part of muscles. Hakkinen et al. (1985) were also among the first to compare electromyography characteristics following a 24-week training program, finding significant improvements in jumping performance. Bobbert. M, made essential contributions to the technical structure of the DJ, finding biomechanical differences, in his first study (Bobbert et al., 1987a), between the DJ with rebounding and the DJ with countermovement, and recommending the former for athletes seeking to improve the mechanical output of the knee extensors and plantar flexors. Nevertheless, his second study (Bobbert et al., 1987b) suggested a FH at 20 or 40 cm when investigating the training effects of rebounding DJ. Bobbert was then the first to scientifically justify a range of FH. (Lees and Fahmi, 1994b), A was the first to refute all previous FH criteria and contributions (Komi and Bosco, 1978b; Bobbert et al., 1987b), indicating that the best performance for a net increase in JH, instantaneous power, and other parameters occurred at 12 cm FH. There have been considerable contributions regarding the best conditions for power production (Pw) (Bosco et al., 1983b; Matic et al., 2015b; Morin et al., 2019), ground reaction forces (GRF), rate of force development (RFD) (Jensen and Ebben, 2007; Ebben et al., 2008; Flanagan et al., 2008; Kossow and Ebben, 2018a), and the reactive strength index (RSI), as well as the differences between CMJ and DJ (Young, 1995; Young et al., 1995; Flanagan et al., 2008; Struzik et al., 2016; Montoro-Bombú et al., 2022), although not always consistent. The emergence of these variables has led to the utilization of different criteria for assuming plyometric training intensity. However, all these parameters require an extensive use of laboratory equipment and analysis, which makes them difficult to implement on a daily basis for plyometric training programs.

In recent years, numerous studies have investigated the effects of plyometric training programs, including: injury prevention and obesity reduction (Nobre et al., 2017; van de Hoef et al., 2019; Zubac et al., 2019; Lee et al., 2020), lower limb power and optimal loading (Miller et al., 2002; Young, 2006; Baechle et al., 2008; Terzis et al., 2016), running economy and, overall performance in dynamic activities (Besier et al., 2001; Balsalobre-Fernandez et al., 2016). This required the development of an effective training program where, we infer, that it was necessary to understand the different components of training, such as exercise volume, frequency, program duration, progression, and intensity. However, a review article (Ramirez-Campillo et al., 2020b) showed that 42% of the studies did not report the intensity of their plyometric training, probably due to methodological difficulties. Furthermore, published research showed no consensus on the best method for determining plyometric exercise intensity (Ebben, 2007; Ebben et al., 2008; Jarvis et al., 2016; Ramirez-Campillo et al., 2020b). On the other hand, although the benefits of plyometrics are well researched, we currently do not find a critical and solid report on the most used variables for the implementation of volume and intensity during such training programs. Therefore, the criteria to determine the main components of plyometric training are an aspect that requires further exploration. Considering the lack of consensus on this problem, it seems justified to systematize the existing criteria for the volume and intensity of plyometric training in the scientific literature. This knowledge may allow more precise monitoring of plyometric training load and provide valuable indications for professionals. Therefore, the purpose of this systematic review was to collate and evaluate the criteria for determining the volume and intensity of drop jumps in the available literature.

## Methods

The study was conducted following the recently updated Preferred Reporting Items for Systematic Reviews and Meta-Analyses (PRISMA) guidelines. (Page et al., 2021; Page et al., 2022).

### Eligibility criteria

Inclusion and exclusion criteria were established *a priori*. Table 1 shows the rigorously determined definitions for type of participants, intervention, type of comparisons, quality of outcomes and study design (PICOS). Randomized studies used a parallel or crossover group design (Spieth et al., 2016; Hariton and Locascio, 2018). These criteria were rigorously followed to reduce the risk of between-group bias and avoid systematic differences in confounding factors (Spieth et al., 2016; Hariton and Locascio, 2018). Therefore, the purpose of this systematic review was to collate and evaluate the criteria for determining the volume and intensity of drop jumps in the available literature.

**TABLE 1:** Inclusion and exclusion criteria.

Guidelines	Inclusion Criteria	Exclusion Criteria
Participants	Athletes between the ages of 16 and 40. Of either sex. Experienced and inexperienced subjects in plyometric training.	People under 16, over 40 and older adults.
Interventions.	It incorporated a PJT program with DJ with rebounding or countermovement. DJ with free arms. Isolated or combined. No overload. Paired control groups or experimental and control groups. Including pretest and posttest.	Rehabilitation programs, horizontal Drop Jump exercise, programs with supplementation (creatine, caffeine, or other), groups that performed DJ with overload or did not report the drop height used during the training program, DJ on unstable surfaces.
Comparisons	Passive or active control group comparisons during a plyometric training program.	Lack of comparisons, comparisons with coordination only, comparisons with change of direction only.
Results	Measurements of vertical jumps, jump height, sprint, acceleration.	Absence of results, not clearly describing the results.
Study designs and article type	Randomized controlled trials incorporating PJT both parallel, crossover, and cluster with more than 4 weeks and no limitation of completion. Original, peer-reviewed research in the English language.	Non-randomized, case reports, cross-sectional, retrospective studies, observational studies. conference abstracts, books and book chapters, published in non-peer-reviewed journals, websites, and preprint editions, non-human studies.

PJT = jump training program; DJ = drop jump.

### Sources information

The searches were carried out from the beginning of the indexing of the databases until the 10th of September 2022. The databases searched were PubMed, SPORTDiscus, Web of Science and, Scopus. Regulatory database records were used. We also contacted experts who provided papers that were not included and met the inclusion criteria. Reference searching of study citations was used as a source of information to detect potentially eligible studies.

### Search strategy

Keyword selection was determined by experts and included: (“Training with adults” OR “Training with athletes” OR “Training with youth” OR “Program with adults” OR “Program with athletes” OR “Program with youth” OR “Effect*") AND (“Vertical Jump” OR “Plyometric*" OR “Drop Jump” OR “Depth jump”). Accounts were created in each database, automatically generating emails for information on new papers. These were received as available and were subject to the review process until the end of the study on the 23rd of December 2022. The search strategies are listed in Table 2.

**TABLE 2:** Search strategy for each database.

Date of the search	8/09/2022	8/09/2022	9/09/2022	10/09/2022
Date base	SPORTDiscus (EBSCOhost)	PubMed/ MEDLINE	WOS	Scopus
Applied database fields used during the search	Title, Abstract,	Title, Abstract	Topic (Title, abstract, author keywords, and Keywords Plus)	Title, Abstract, keyword
Restrictions for the search	None			
Examples of the strategy PubMed/ MEDLINE	("Plyometric"[Title/Abstract] OR “Depth Jump” [Title/Abstract] OR "Drop jump"[Title/Abstract] OR "Vertical jump"[Title/Abstract] OR "Jump*"[Title/Abstract] OR "Stretch shortening cycle"[Title/Abstract] OR "Stretch shortening cycle"[Title/Abstract]) AND ("Training"[Title/Abstract] OR "Training program"[Title/Abstract] OR "Training effect*"[Title/Abstract] OR "Training intervention"[Title/Abstract])			
Examples of the strategy SPORTDiscus	TI (“Plyometric” OR “Depth Jump” OR “Drop Jump” OR “Vertical Jump” OR “Jump*” OR “Stretch-shortening cycle” OR “Stretch shortening cycle”) AND TI (“Training” OR “Training Program” OR “Training Effect*” OR “Training intervention”)			
	AB (“Plyometric” OR “Depth Jump” OR “Drop Jump” OR “Vertical Jump” OR “Jump*” OR “Stretch-shortening cycle” OR “Stretch shortening cycle”) AND AB (“Training” OR “Training Program” OR “Training Effect*” OR “Training intervention”)			
Examples of the strategy Web of Science	“Plyometric” OR “Depth Jump” “Drop Jump” OR “Vertical Jump” OR “Jump*” OR “Stretch-shortening cycle” OR “Stretch shortening cycle” (Topic) AND “Training” OR “Training Program” OR “Training Effect*” OR “Training intervention” (Topic)			
Examples of the strategy Scopus	(TITLE-ABS-KEY ("Plyometric" OR "Depth Jump" OR "Drop Jump" OR "Vertical Jump" OR "Jump*" OR "stretch-shortening cycle" OR "Stretch shortening cycle") AND TITLE-ABS-KEY ("Training" OR "Training Program" OR "Training Effect*" OR "Training intervention"))			

### Selection process

Two authors (RM and CB) developed the search process, removing duplicates using reference management software (EndNote TM X9, Clarivate Analytics, Philadelphia, PA, United States). They then performed title and abstract review and full-text analysis independently. By agreement between the two, studies in doubt of acceptance were separated for discussion, which was discussed and agreed upon with 100% agreement.

### Data collection process and data items

The authors developed the data using criteria from other reviews (Ramirez-Campillo et al., 2018a; Ramirez-Campillo et al., 2020a). They were defined *a priori* and included the following information: 1 = name of authors and study; 2 = sport or activity; 3 = number of subjects, age (years), and sex; 4 = if the methodology states a plyometric training program; 5 = if the methodology states heights criteria before performing the exercise; 6 = if the program includes other jumps; 7 = if the methodology states the heights selected to execute the drop jump; 8 = if it presents volume parameters by weeks or sessions; 9 = if the methodology declares criteria for volume variation; 10 = training frequency and a total number of sessions (days/week); 11 = recovery between exercises (seconds or minutes); 12 = recovery between sets (seconds or minutes); 13 = presents the type of work surface (asphalt, grass, and others.); 14 = if the study presents cross-training and a total number of sessions (days/week)); 15 = if the study presents crossover; and 16 = Increase in performance shown by the program (Eq. (1)). A data extraction sheet was developed and tested with ten randomly selected studies, adapted from the Cochrane Consumers and Communication Group’s data extraction template (https://cccrg.cochrane.org/cochrane-author-resources).

$$Pi\left(\%\right)=\frac{\left(Cr-\mathrm{Pr}\right)*100}{\mathrm{Pr}}$$ (1)

Where: Pi (%) = Performance increase.

Pr = Previous result.

Cr = Current result.

### Studies risk of bias assessment

The methodological quality of the chosen studies using the recommended language for sports science (Winter et al., 2016) was assessed using the Grading of Recommendations, Assessment, Development and Evaluation (GRADE) model (Aguayo-Albasini et al., 2014). This model is advocated for RCTs not only because of the strength of the recommendations, but because it is based on the quality of the studies, risk balance, etc. According to its recommendations, the quality of evidence is classified as high, moderate, low, and very low. To individualize the level of recommendation and methodological quality, a specific modification of this scale was made for this study. This consisted of reporting individual studies only, thus excluding group studies, on the same variable (Guyatt et al., 2011).

## Result

**FIGURE 1:**
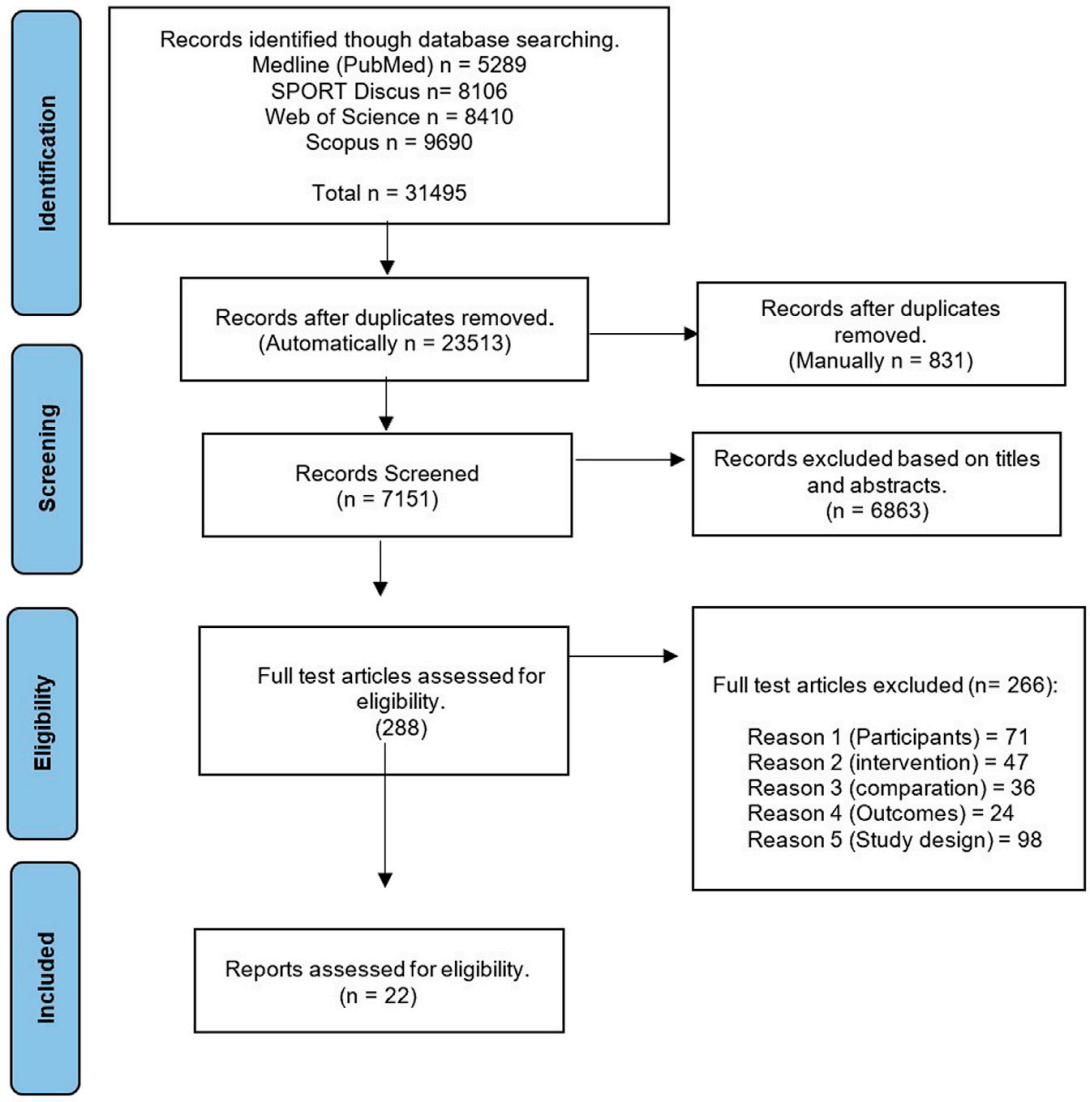
Prism flow chart on the results of the literature search.

A total of 31495 studies were identified in the databases and exported to the bibliographic reference management software (EndNote TM X9, Clarivate Analytics, Philadelphia, PA, United States). A total of 23513 studies were automatically removed as duplicates, and a further 831 studies were manually removed as duplicates. The remaining studies (7151) were screened by title and abstract, taking into account the inclusion and exclusion criteria and then their relevance, resulting in the elimination of further 6863 studies. A total of 288 studies were eligible for full-text review. Once the full-text review was completed, 266 studies were excluded based on the following criteria: participants (71), intervention (47), comparison (36), outcomes (14), and study design (98). Thus, a total of (22) were included in the review for critical review (Figure 1).

### Quality assessment and risk of bias of the studies chosen for the review

According to the GRADE assessment (Table 3), the study quality was average, with only one study that we considered to be of high quality. Applying the intraclass correlation index, it was found that the independent reviewers involved in the eligibility had a reliability of 0.89.

**TABLE 3:** Application of the GRADE scale for the RCTs included in the study.

	Quality assessment				Results summary			
					n-subjects		Absolute risk	
Studies	1	2	3	4	5	6	7	8
(Lum et al., 2022)	No pause between rep.	M	JH	D	n=22	n=11	d=0.48	Mod.
	No evaluation of Int.							
	No volume variation criterion							
(Falch et al., 2022)	No pause between rep.	P	RSI	D	n=21	n=10	d=0.68	Bajo
	Insufficient volume							
	No volume variation criterion							
(Sanchez-Sixto et al., 2021)	No assessment of Int.	P	JH	D	n=36	n=11	d=0.5	Mod.
	No volume variation criterion							
	Does not report variation in reported heights.							
(do Carmo et al., 2021)	No assessment of Int.	P	JH DJ	P	n=28	n=15	d=0.56	Mod
	No volume variation criterion							
(Ahmadi et al., 2021)	No assessment of Int.	D	RSI JH	D	n=17 n=17	n=9 n=9	d=0.43 d=0.75	Mod.
	No volume variation criterion							
(Laurent et al., 2020)	No assessment of Int.	D	JH	D	n=32	n=32	d=0.75	Mod
	No volume variation criterion							
(di Cagno et al., 2020)	No assessment of Int.	D	JH CMJ	P	n= 54	n=28	d=0.27	Bajo
	No volume variation criterion							
(Jeffreys et al., 2019)	No volume variation criterion	D	RSI 45cm	D	n=36	n=10	d=0.27	Mod
(Bogdanis et al., 2019)	No assessment of Int.	D	RSI	D	n=15	n=8	d=0.12	Mod
	No volume variation criterion							
(Bianchi et al., 2019)	No assessment of Int.	P	(sprint 30 m)	P	n=21	n=11	d=0.4	Bajo
	No volume variation criterion							
	It does not report on the variation of reported heights.							
(Ramirez-Campillo et al., 2018d)	It does not report on the variation of reported heights.	N	JH	N	n=23	n=16	d=0.57	Alto
(Terzis et al., 2016)	No assessment of Int.	D	JH CMJ	P	n=20	n=10	d=0.66	Mod
(Ramírez-Campillo et al., 2014)	No assessment of Int.	D	JH CMJ	P	n=36	n=36	d=0.48	Mod
(Chelly et al., 2014)	No assessment of Int.	D	JH CMJ	D	n=23	n=12	d=1.00	Mod
(Ramírez-Campillo et al., 2013)	No assessment of Int.	D	JH CMJ	P	n=29	n=8	N/M	Mod
(Khlifa et al., 2010)	No assessment of Int.	D	JH CMJ	P	n=27	n=9	d=2.60	Mod
	No volume variation criterion							
(Sedano et al., 2009)	No assessment of Int.	D	JH DJ	D	n=20	n=10	d=0.77	Mod
	No volume variation criterion							
(de Villarreal et al., 2008)	No assessment of Int.	D	JH DJ 40	N	n=42	n=10	d=‐‐‐	Mod
(Markovic et al., 2007)	No assessment of Int.	D	JH DJ 30	N	n=93	n=30	d= 0.90	Mod
(Luebbers et al., 2003)	No assessment of Int.	D	JH CMJ	P	n=38	n=19	d=0.37	Bajo
(Gehri et al., 1998)	No assessment of Int.	D	JH CMJ	D	n=28	n=11	d=0.22	Mod
	No volume variation criterion							
(Chelly et al., 2010)	No assessment of Int.	D	JH CMJ	D	n=23	n=12	d=0.33	Mod

1 = Program limitations; 2 = Program inconsistencies; 3 = Criteria for improvement; 4 = Bias; 5 = Total number of subjects; 6 = Total number of subjects intervened with DJ; 7 = Effect size found; 8 = GRADE quality; *working sessions, not weeks. **after four weeks of work. D = Disappeared; P = Present and N = Not present.

### Study characteristics

The RCT included in this review recruited groups of physical education students and recreationally active individuals (*n* = 8), moderately trained cross-country athletics groups (*n* = 4), national level Football groups (*n* = 4), national level Handball groups (*n* = 2), national level Basketball groups (*n* = 2). The remaining groups were Rugby, Fencing, Volleyball and untrained subject. This amounted to 686 recruited individuals, of which *n* = 337 were intervened with DJ and DeJ training. The mean age of all participants was 25.79 ± 4.76 years, but it is worth noting that one study (Luebbers et al., 2003) did not present the age of the participants, only reporting that they were university students. Although it can be inferred that they were between 18 and 25 years old, the study was excluded from the calculation of the mean age. Of the 22 included RCT, six groups presented results with women only, 15 presented results with men only, and the remaining 4 presented a mixed study. The duration of plyometric training programs ranged from 4 to 12 weeks, while training frequencies were between 1 and 4 times per week. Twenty-one of the 22 included studies did not present a selection criterion for individual FH in the procedure. Twenty-one of the 22 included studies standardized the FH, with ranges between 20 and 60 cm. We also found that the total jump per session ranged between 10 and 140. However, all the 22 included studies did not present a procedure criterion for the selection of the plyometric work volume. Thus, 18 of the included studies present a variation of the training volume without methodological justification, and the remaining seven do not explain why they maintain the same work volume during all the weeks of training. The type of landing surface is not reported in 10 studies, and two others are considered unclear. The objectives and main results of the included studies are detailed in Table 4, while Table 5 shows the general characteristics of the studies in this systematic review.

**TABLE 4:** The objectives and main results of the included studies.

Studies	Objective	Main Results
(Lum et al., 2022)	To compare: Dynamic versus isometric strength- effect on JH.	Both plyometric and isometric exercises improved JH.
(Falch et al., 2022)	To investigate: Strength vs plyometric exercises on change of direction.	Both groups improved, but strength training was effective, and plyometric training was not sufficient.
(Sanchez-Sixto et al., 2021)	To compare: PJT vs combined PJT on the kinetics and kinematics of the vertical jump.	Combined PJT could provide better results in jumping performance than PJT alone.
(do Carmo et al., 2021)	To evaluate: PJT vs stimulation To verify: PJT vs perceived exertion, running pace and affective feeling.	PJT shows no change in stimulation. PJT shows no change in rhythm, perceived effort, and affective feeling.
(Ahmadi et al., 2021)	To evaluate: PJT on sand vs hard surface on the biomechanical variable.	PJT on hard surface had better results for biomechanical variables than sand surface.
(Laurent et al., 2020)	To compare: PJT DJ with flexed and extended knees on Achilles tendon stiffness and JH.	PJT increased JH in both groups, but JH of DJ20 cm and tendon stiffness showed better results for the group with extended knees.
(di Cagno et al., 2020)	To evaluate: PJT using a device vs PJT on range of motion, explosive and reactive strength.	PJT for both groups show no improvement in lunge distance, explosive and reactive strength
(Jeffreys et al., 2019)	To identify: Effectiveness of high and low volume plyometric loads on the stretch-shortening cycle.	Low volume PJT elicited the same improvement in RSI performance as a high-volume program.
(Bogdanis et al., 2019)	To compare: Unilateral versus bilateral PJT on maximal strength and rate of force development.	Unilateral PJT shows better results in strength and rate of force development for each leg individually.
(Bianchi et al., 2019)	To compare: High and low volume PJT on jumping, sprinting and change of direction abilities.	Both high and low-volume PJT induce benefits in jumping, sprinting and change of direction tests.
(Ramirez-Campillo et al., 2018d)	To compare: PJT with one session versus two sessions per week at the same weekly volume on fitness components.	PJT with one or two sessions has no additional effects on fitness development when the jumping volume is matched.
(Terzis et al., 2016)	To investigate: Effects of low intensity running vs power training on power development.	PJT has better results in JH and maximal power. Maximal isometric strength, rate of force development and maximal strength.
(Ramírez-Campillo et al., 2014)	To examine: Short-term PJT on explosive strength and endurance.	PJT improved running time, CMJ and DJ performance.
(Chelly et al., 2014)	To determine: Substitution of short-term PJT for some existing exercises would improve explosive movements.	Bi-weekly PJT improves important components of handball performance.
(Ramírez-Campillo et al., 2013)	To examine: Short-term PJT with different volumes and surfaces on neuromuscular performance.	High-volume PJT improves explosive performance, and PJT on hard surfaces increases explosive performance.
(Khlifa et al., 2010)	To examine: loaded versus unloaded PJT on vertical jump.	PJT with weights presents better results on vertical jumps.
(Sedano et al., 2009)	To examine: PJT on explosive strength, kicking speed and body composition.	PTJ improves explosive strength and kicking speed.
(de Villarreal et al., 2008)	To examine: PJT vs different frequencies and volumes on maximal strength, vertical jump and running.	PJT frequency two moderate volume sessions produce similar improvements at a greater efficiency than high volume frequency 4. Also, similar results in strength, jumping and running.
(Markovic et al., 2007)	To evaluate: PJT versus speed training on muscle function and dynamic athletic performance.	Sprint training produces similar or even more significant effects on muscle function and dynamic performance.
(Luebbers et al., 2003)	To determine: PJT of 4 vs seven weeks, followed by four weeks without plyometric training, on vertical jump performance and anaerobic power.	The four- and seven-week plyometric programs are equally effective in improving vertical jump height and anaerobic power after a 4-week recovery period.
(Gehri et al., 1998)	To determine: PJT of different techniques versus vertical jump capacity, positive energy production and elastic energy utilization.	Neither technique improved elastic power. In activities involving stretch-shortening cycles, DJ training was superior to CMJ.
(Chelly et al., 2010)	To examine: Combined hurdle and depth jump PJT on maximal power, strength, JH and muscle volume.	Biweekly PJT improved components of athletic performance relative to standard in-season training.

**TABLE 5:** Characteristics of the RCTs included in the study.

Studies	1	2	3	4	5	6	7		8	9	10	11	12	13	14
							weeks	vol							
(Lum et al., 2022)	Athletics endurance	n=22 e= 37±6 s= mix	No	No	DJ +	40, 50 y 60 cm	1 2-5 6	3 x 5 4 x 5 2 x 5	No	6 weeks. 2 x W Total: 18	no	3	No	No	9.20 % (JH)
(Falch et al., 2022)	Handball	n=21 e= 17±2 s= Fem	No	RSI	DJ +	40 cm	1 2 -3 4 5 6 7	1 x 6 1 x 8 1 x 10 1 x 12 1 x 8 1 x 5	No	8 weeks. 1 o 2 x W Total: 12	no	>2	Contact mat	No	37.0 % (RSI)
(Sanchez-Sixto et al., 2021)	Basketball	n=36 e= 23±6 s= Mal	No	No	DJ	20, 25, 30 y 40 cm	1-3 4 5 6	3 x 6 3 x 4 4 x 4 3 x 3	No	6 weeks. 2 x W Total: 12	1	3	No	No	5.71 % (JH)
(do Carmo et al., 2021)	Recreational runners	n=28 e= 33,3 s= Mal	No	No	DeJ +	40 cm	4-5 6 7 8	2 x 6 3 x 6 4 x 6 2 x 6	No	8 weeks 2 x W Total: 16	-	-	No	No	7.28 % (JH DJ)
(Ahmadi et al., 2021)	Volleyball	n=17 e= 23±2 s= Fem	No	No	DeJ +	40 cm	1-8	2 x 6	No	8 weeks 2 x W Total: 16	5 to 15´´	30 to 60´´	Wood vs sand	No	13.6 % (JH) 11.5 % (RSI)
(Laurent et al., 2020)	Physically active	n=32 e= 22.5 s= Fem	No	No	DJ +	20, 40 y 60 cm	1 2 3-10	3 x 10 4 x 10 5 x 10	No	10 weeks 2 x W Total: 20	2´	1.5 to 3´	Ground	No	13.7 % (JH)
(di Cagno et al., 2020)	Fencing	n=54 e= 17.5 s= Mal	No	No	DJ	50 cm	1-2 3-4 5-6	1 x 7 2 x 7 3 x 7	No	6 weeks 2 x W Total: 12	2´		Ground	No	4 % (JH) CMJ
(Jeffreys et al., 2019)	Rugby	n=36 e= 20±1 s= Mal	No	RSI	DJ	individual	2-6	2 x 5	Does not vary	4 weeks 2 x W Total: 8	-	-	No	No	15.78 % (RSI)
(Bogdanis et al., 2019)	Moderately trained	n=15 e= 19.6 s= Mix	No	no	DJ +	30 cm	1-3 4-6	2 x 10 3 x 10	No	6 weeks 2 x W Total: 12	-	1	No	No	4.30 % (RSI)
(Bianchi et al., 2019)	Football	n=21 e= 17±8 s= Mal	No	No	DJ +	60 cm	1-8	4 x 5	Does not vary	8 weeks 2 x W Total: 16	-	-	No	No	1.43 % (Sprint 30 m)
(Ramirez-Campillo et al., 2018d)	Football	n=23 e= 21±3 s= Fem	No	No	DJ +	5-35cm	1-2 3-4 5-6 7- 8-	5 x 16 5 x 20 5 x 24 5 x 28 5 x 14	No	8 weeks 2 x W Total: 16	5-15´´	30-60´´	Assorted grass and ground	No	
(Terzis et al., 2016)	Physical Education students	n=22 e= 20±2 s= Fem	No	No	DJ +	20, 30 y 40 cm	1-6	8 x 3	No	6 weeks 1 x W Total: 6	30´´	1´	Contact mat	No	10.90 % (JH CMJ)
(Ramírez-Campillo et al., 2014)	Athletics endurance	n=36 e= 22.1 s= Mix	No	No	DJ +	20, 40 y 60 cm	1-6	6 x 10	Does not vary	6 weeks 2 x W Total: 12	15´´	2´	Wood	No	8.86 % (JH CMJ)
(Chelly et al., 2014)	Handball	n=23 e= 17.2 s= Mal	No	No	DJ +	40 cm	5-8	4 x 10	Does not vary	9 weeks 2 x W Total: 18	5´´		No	No	9.52 % (JH CMJ)
(Ramírez-Campillo et al., 2013)	Physical Education students	n=29 e= 16.86 s= Mal	No	No	DJ	20, 40 y 60 cm	1-6	6 x 10 12 x 10	Does not vary	7 weeks 2 x W Total: 14	5´´	1,5´	Wood OR mattress (3 cm)	No	N/M (JH CMJ)
(Khlifa et al., 2010)	Basketball	n=27 e= 23.61 s= Mal	No	No	DJ +	40 cm	1 2 3-10	3 x 5 5 x 9 6 x15	No	10 weeks 3 x W Total: 30	15 a 30´´	-	No	No	7.0 %(JH CMJ)
(Sedano et al., 2009)	Football	n=20 e= 22.9 s= Fem	No	No	DJ +	50 cm	1-6 2-4 3-5 8-10 9-11 12	8 x 5 10 x 5 9 x 5 11 x 5 10 x 5 9 x 5	No	12 weeks 1 x W Total: 12	1´	4´	Synthetic hard floor	No	18.0 % (JH DJ)
(de Villarreal et al., 2008)	Physical Education students	n=42 e= 23.5 s= Mal	No	No	DJ	20, 40 y 60 cm	1-7	6 x 10	Does not vary	7 weeks 4 x W Total: 28	-	1´	3 cm mattress	No	18.78 % (JH JD)
(Markovic et al., 2007)	Physical Education students	n=93 e= 20.1 s= Mal	No	No	DJ +	40 cm	8-11	4 x 10	No	4 weeks 3 x W Total: 12	5´´	-	Athletics track	No	14.2 % (JH JD)
(Luebbers et al., 2003)	Physical Education students	n=38 e= University s= Mal	No	No	DeJ +	40 cm	3 4 5-6 7	2 x 10 3 x 10 6 x10 7 x 10	No	5 weeks 3 x W Total: 15	15-30´´	-	No	No	4.0 % (JH CMJ)
(Gehri et al., 1998)	Recreationally active	n=28 e= 20.3 s= Mix	No	No	DJ	40 cm	1-2 3-10	2 x 8 4 x 8	No	10 weeks 2 x W Total: 20	5´´	1´	No	No	0.69 % (JH CMJ)
(Chelly et al., 2010)	Football	n=23 e= 19±0.7 s= Mal	No	No	DJ +	40 cm	5-8	4 x 10	Does not vary	9 weeks 2 x W Total: 18	5´´		Grass	No	2.43 % (JH CMJ

1 = Sport or activity; 2 = Number of subjects, age and gender; 3 = Methodology states a plyometric preparation program; 4 = Methodology states height criteria before performing the exercise; 5 = Program includes DJ, DeJ plus other jumps; 6 = Methodology states the height selected to perform the DJ or DeJ; 7 = Volume structure by weeks or sessions; 8 = Methodology states criteria for volume variation; 9 = Frequency of training and the total number of sessions; 10 = Recovery between exercises; 11 = Recovery between sets; 12 = Type of work surface; 13 = The study presents crossover; and 14 = Increase in performance shown by the Program.

## Discussion

The purpose of this systematic review was to collate and evaluate the criteria for determining the volume and intensity of drop jumps within training programs, according to the available literature. The review protocol was registered on the International Platform of Registered Systematic Review and Meta-Analysis Protocols available at https://inplasy.com/inplasy-2021-2-0051/. So far, this is the first systematic review that analyze the literature regarding the volume and intensity of DJ and DeJ in plyometric training. In opposite to other scoping reviews (Ramirez-Campillo et al., 2020c) this is critical and propositional. Under the guidance of Boolean operators (AND and OR), this review found a total of 31495 studies in different databases, which were processed to finally include 22 studies in our review. The results reveal that, for plyometric training, there is still a lack of consensus in determining the volume and intensity of exercises. There is a need to deepen the quality of research processing in order to clearly determine volume and intensity of plyometric training, thus its effect. Therefore, in the present discussion, we aim to address the discordance within the literature by developing operational frameworks that facilitate the understanding and design of training programs. Taking into account variables such as the athletes’ experience level, along with training volume, intensity, density, duration, weekly frequency, exercise type and execution criteria, as well as a practical rationale for implementation.

### Main criticisms and observations regarding training programs

Most of the selected studies showed a limited knowledge of the main characteristics of plyometric training. In fact, 90.90% (*n* = 20) of the reported study designs do not specify some variables that greatly affect the training effect (Bompa and Buzzichelli, 2019). For example, for what concern the landing surface, most studies do not report either the main objective of the plyometric work or the criteria of selection for the surface type. It is known that the surface type influences the stretch-shortening cycle (Bobbert et al., 1987a; Ramírez-Campillo et al., 2013) and that soft surface, such as grass or sand, do not allow contact times to be minimized, thus increasing reaction times. Hard surfaces on the other hand, allow shorter contact times, which guarantees a better reactivity (Ramírez-Campillo et al., 2013). These criteria are sometimes reported with a lack of clarity (de Villarreal et al., 2008; Sedano et al., 2009) or are contradictory to the stated objective (Chelly et al., 2010), but one study is quite clear with its statement related to the type of surface (Ramirez-Campillo et al., 2018d). Considering that the type of surface is closely related to the training effects of plyometrics, not stating the surface used in a study can lead to problems both in replicating it and in interpreting its results (Ramírez-Campillo et al., 2013; Ramirez-Campillo et al., 2020c). None of the included studies analyses the timing of utilization of hard and soft surfaces within the athletes’ preparation phase. Although we can clearly infer the best time to use with different surfaces from the results of some studies (Ramírez-Campillo et al., 2013; Arazi et al., 2014; Ahmadi et al., 2021; Jacobs et al., 2021; Lannerstrom et al., 2021; Pereira et al., 2022). It would be interesting for future research to identify which type of surface can be the best choice according to the phases of the annual plan (general preparation, specific preparation, or competition phase).

Previous studies (Ramirez-Campillo et al., 2015), have indicated that plyometric training programs combined with other strength training means have better results than plyometric training alone. Although logical and very clear, this reasoning could be considered a tautology that needs to be reanalyzed. The combination of DJ training with strength training (Sanchez-Sixto et al., 2021), can present improvements in the kinetics and kinematics of the vertical jump, but this does not necessarily mean that 100% of the DJ training goals or 100% of the strength training goals have been met, and it would be implausible to determine the contribution of each one to the end result. The same way, when combinations of DJ, CMJ, SJ and multiple other jumps are performed (Table 5), although this is known to contribute to the corresponding training adaptations (Ramírez-Campillo et al., 2015; Ramirez-Campillo et al., 2015), researchers are uncertain to what extent each exercise affected the outcome. In this sense, while acknowledging the contributions of mixed plyometric studies, this research encourages the return to studies where the effects on performance variables of plyometric training, as a whole and in its components, can be isolated. Another fundamental element to be considered, is the population of such research studies. Several studies report implementing plyometric training programs with recreationally active individuals, physical education students or athletes with no previous experience in plyometric training (Table 5). In all these cases, care should be taken in interpreting the results: as in any activity performed with untrained subjects, if injuries do not occur, positive effects are most likely to be expected. Conversely, we can presume that when working with a population composed of experienced athletes, it may be difficult to obtain large effect sizes and large statistically significant differences due to the high level of specific work accumulated over the years.

An important variable that, in our opinion, is under-recognized, is the density of plyometric training. The included studies only refer sparingly to the weekly frequency of work, and none study indicates the density of the session. Density can be interpreted as the ratio between the total duration of a session, and the actual work performed (Bompa and Buzzichelli, 2019); for that reason, rest intervals become a determinant of the session density. Consequently, during plyometric training, the longer the recovery time, the lower the training density. This variable should also be considered when appraising training intensity, as it could be affected in the actual intensity in terms of power output (Lawton et al., 2006).

Another element that is too often overlooked, is the identification of the participants’ level of experience. A previous report (Ramirez-Campillo et al., 2020c) had already shown that dichotomous variables (yes or no) do not gather sufficient information about athletes. We note that other studies (Sanchez-Sixto et al., 2021) report the athletes’ years of training experience, but they do not specify whether the individuals came from the practice of team sports, power, or endurance sports, nor is it clear whether the experience (years) in plyometric training is systematic or just occasional. These criteria may be necessary to identify *a priori* whether large effect sizes can be expected at the end of the training interventions, as more experienced subjects need training means of higher specificity and intensity to deliver comparatively minimal, yet important, training effects. There is clearly an urgent need to standardize these elements.

As a consequence, although we recognize that experience is a multidimensional factor, we agree that there are general characteristics that mediate the influence of experience. Based on recommendations from previous reviews and meta-analyses (Moran et al., 2019; Ramirez-Campillo et al., 2020c; Moran et al., 2021; Ramirez-Campillo et al., 2021; Clemente et al., 2022), we propose five elements that we consider helpful in highlighting the participants’ level of experience in plyometric and ballistic activities: (A) The type of sport population. This is related to the level of involvement in physical activity and sports. (B) The type of sport practiced. This is determined by the performance characteristics of the sport practiced and its relationship with plyometric actions. (C) Time of experience with either systematic or interspersed practice of plyometric exercise. (D). The level of the participant’s integral reactive strength, and (E) the height reached in a CMJ. All these parameters were modified and adapted to the context following previous recommendations (Swann et al., 2015) and are shown in Table 6. Although it is beyond the scope of this review to list all the possible sporting activities for each level, this table can be a crucial tool to standardize the criteria to assess and state the athletes’ level of experience in new research.

**TABLE 6:** Methodological consideration for the determination of participants' experience in plyometric and ballistic activity.

Variables/ points	5	4	3	2	1
A. Type of population	International participation in competitions	Participation in national competitions	Participation in regional competitions	Systematic recreational sports practice	Sedentary or occasional sporting activity
B. Type of sport	Maximum, explosive, and reactive strength	Team sports.	Combat sports	Endurance, artistic, and Water sports	Random practice of team or individual sports, or no practice
C. Time experience in plyometrics	≥ 6 years	5-4 years	3-2 years	≤ 1 year	Never
D. IRSI 40 cm	≥ 1.75	1.74–1.25	1.24-1.00	0.99-0.50	≤ 49
E. CMJ-JH	≥ 50 cm	49-42 cm	41-34 cm	33-21 cm	≤ 20
Level of plyometric experience	Level 4 = ≥20 Long and systematic experience of extensive and intensive plyometric exercise	Level 3 = 13–19 Experience in plyometric development	Level 2 = 7–12 Basic plyometric experience	Level 1 = 1–6 Low introductory experience	Level 0 = ≤ 1 No Experience
Equation	Participant's level of plyometric experience* = [(A+B+C/2) /3] x (D+E)*Individualized criteria				

IRSI = integral reactive strength index; JH = jump height; CMJ = countermovement jump.

Individualization of training is considered one of the fundamental principles of the sports training process (Bompa and Buzzichelli, 2019). However, individualization is not factored in by the researchers of most of the included studies. Only two studies (Ramirez-Campillo et al., 2018d; Jeffreys et al., 2019) reported individualizing the heights of fall, but still presented problems with the individualization of volume and intensity parameters. Likewise, the studies with equaled volume and FH (Table 5) present significant performance increases in the results but with great inter-individual outcome differences. For instance, 40 cm of height may represent 70% of the jumping capacity for one subject, but 90% for another. Similarly, a pre-set volume of, for example, 4 sets of 10 repetitions, may be optimal to improve a subject’s power output while for another subject it could represent a power-endurance type of work, thus eliciting very different adaptations, not in line with the scope of the intervention. Although one study (Ramirez-Campillo et al., 2018c) considers plyometric individualization impractical for team sports, we considered it an essential element when working with high-performance sport.

### Critical discussion on the use of the fall height as a determinant of intensity

Several authors emphasize that the FH is the sole determinant of the DJ and the DeJ’ intensity. Yet, FH alone does not generate intensity and needs to be related to a performance variable associated with the exercise. Based on this systematic review, we understand that plyometric training intensity is determined by the association of a quantifiable variable (e.g., power output, impulse, RFD, reactive strength, vertical height or horizontal distance, ground reaction forces, load index, electromyographic activity, etc.) to a previously determined FH. In this sense, we can have scenarios with different intensity criteria for the same FH. E.g., given a DJ50, if we compare an athlete (a) who displays a concentric GRF of 4298 N, 3.15 of RSI, and a power output of 6452 W, to another (b) who has 3954 N of concentric GRF, 2.54 of RSI and 6462 W of power, it is clear how they may require different FH for maximizing concentric force production and reactive strength, but the same FH for maximum power output.

**FIGURE 2:**
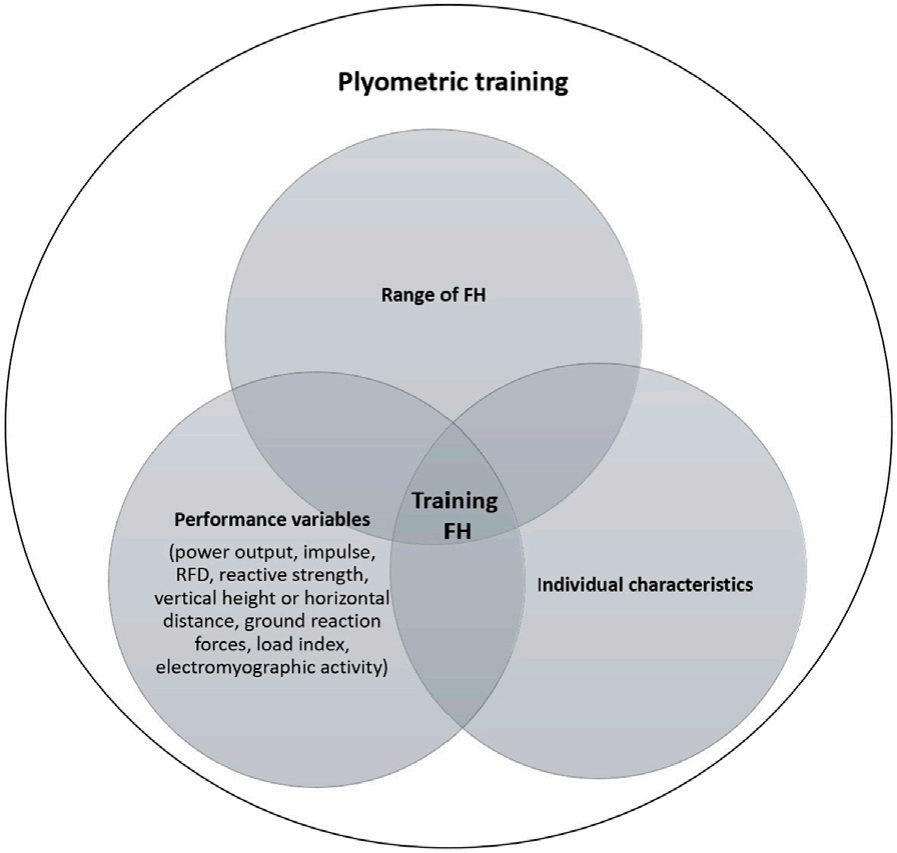
The determination process for the optimal training FH.

FH and its possible effects have been extensively addressed in the literature (Komi and Bosco, 1978a; Bobbert et al., 1987b; Matic et al., 2015a), but with still somewhat inconsistent results. The literature reports that the range of FH can be between 0.12 and 0.80 (Lees and Fahmi, 1994a; Viitasalo et al., 1998), although we know that higher FH have been studied (Clutch et al., 1983). Still, this can be considered a too wide FH range to be integrated in the training program of an athlete, given a training effect goal in a period of time. Therefore, using such a wide range of FH in a plyometric training program can be considered a methodological error. For plyometric training, we recommend that coaches consider a range of optimal FH, which should be related to the plyometric goal to be achieved, as shown in Figure 2.

In a training program, the FH contributes to training specificity (Ebben and Petushek, 2010) and enhances the magnitude of the different adaptations (Young, 1995). FH in excess of the individual capacity led to prolonged eccentric loading, which is synonymous with excessive plyometric loading. This causes longer GCT, which possible partially dissipate the accumulated elastic energy, thus preventing maximum jump height (Asmussen and Bonde-Petersen, 1974). Another element to consider when selecting the fall height is the principle of specificity. According to this principle, the way the athlete performs the plyometric activity will determine the training effect (Walshe et al., 1998). Furthermore, the higher the FH, the higher - up to a certain limit - the levels of neuromuscular pre-activation (Taube et al., 2012; Di Giminiani et al., 2020), the higher the speed that can be achieved in the eccentric phase, and thus the higher the contractile potentiating mechanisms of the muscle spindles and the elastic energy contribution by the tendons (Flanagan and Comyns, 2008). These mechanisms also depend on the individual’s strength level, associated with maintaining active stiffness during the movement. All these conditions created by the increase or decrease in fall height are what can subsequently influence the intensity factor. Likewise, two athletes dropping from the same fall height may not be training at the same relative intensity (Byrne et al., 2017).

Although the literature recognizes the criteria discussed above, in this review, 90.99% of the included studies (Table 5) present arbitrary criteria for selecting the FH. Their procedures can be defined “arbitrary”, as there are no *a priori* assessment criteria to determine which heights are to be used according to the individual characteristics of the subjects. In addition, there is no clear information regarding the relationship between the selected heights and the intervention goals for each subject. Some authors have recommended to limit plyometric exercises to heights of less than 40 cm, since they found that at heights between 40 and 60 cm, there was a reduction in propulsive power, vertical impulse and reactive jump height (Bobbert, 1990; Lees and Fahmi, 1994b; Voigt et al., 1995; Peng, 2011). However, this recommendation is highly questionable, as we consider that it cannot be generalized to comprise all types of population, including, for instance, high-level power athletes, who routinely use higher fall heights in their training programs (Kobal et al., 2017).

## General discussion on plyometric intensity

Intensity is one of the most complex concepts within the theory and methodology of plyometric training. There is still no definitive consensus among researchers about the determination of plyometric training intensity, making it harder to be defended. In order to understand the multidimensional value of plyometric intensity, we need to revise the results of the most current studies on the topic.

It has been stated that plyometric intensity depends on the amount of tension exerted on muscles, connective tissue and joints (Kossow and Ebben, 2018b). Based on this statement, a DJ from 70 cm may not be considered high intensity, if the muscle actions are not performed to the maximum, or, on the contrary, it may be considered excessive intensity, if they are inhibited due to too high eccentric loading (Ruan and Li, 2010). Hence, intensity should be directly associated with a specific metric and not only with the FH. On this topic, research has suggested that intensity can be examined based on different kinetic parameters (Ebben et al., 2008), others made contributions by analyzing the relationship between fall height and intensity (Bobbert et al., 1987b; McNitt-Gray, 1993; Young et al., 1999; Walsh et al., 2004; Wallace et al., 2010). For example, one study (Bobbert et al., 1987b) found no significant biomechanical differences in the joints during the eccentric phase of a DJ20 and a DJ40, but the eccentric phase maximum power was lower in the DJ60 than in the DJ40, and the DJ60 and DJ20 had similar values. Another study (Wallace et al., 2010) has found that eccentric GRF increases are directly proportional to the FH. This finding is likely due to gravity’s uniform acceleration and the landing strategies of the subjects. If the athletes consciously hit the platform at landing, the eccentric forces will increase considerably. Our recommendation for high-performance athletes is to evaluate the GRF of the concentric phase, which can hardly be influenced by other variables other than the purely concentric forces of the athletes. Considering these parameters, we cannot disregard the power output as a significant plyometric intensity value (Di Giminiani and Petricola, 2016; Morin et al., 2019).

On the other hand, although the EMG is criticized for its lack of practicality in applied settings (Ramirez-Campillo et al., 2020a), some studies consider it the most important way of assessing plyometric intensity (Kossow and Ebben, 2018b). This is consistent with several studies that found higher EMG activity when performing a DJ60 and lower activity with a DJ20 (Peng et al., 2011), attributing a higher level of intensity for the DJ60. Plyometric exercises performed with a flexion at the knee joint, show higher EMG activity compared to rebounding exercises with no flexion at the knee joint (Jarvis et al., 2016). The same group of researchers evaluated the vastus lateralis and biceps femoris muscle activity during the concentric phase of the CMJ and the DJ. No significant differences were found for these muscle groups during both exercises. This could indicate that these two exercises, for what concern fibers recruitment during the concentric action, do not present significant statistical differences in intensity, yet it does not mean that they produce the same jump performance, due to the difference in elastic energy storage (Ishikawa et al., 2006). These results are consistent with other criteria previously investigated (Wallace et al., 2010; Sugisaki et al., 2013). With a different view, studies have been criticized for classifying plyometric exercises into low, medium and high intensity (Fowler and Lees, 1998; Potach and Chu, 2008). Although this classification (based on a gradual increase in intensity level) seems to make logical sense, research showed different results in lower limb mechanical performance depending on the type of plyometric exercise (Sugisaki et al., 2013). This is also reflected in other studies examining the EMG (Kossow and Ebben, 2018b). Previous research has showed that EMG activity for the Tuck Jump is highest for the muscles around the ankle. At the same time, only moderate levels of the neural drive are present for the muscles around the knee. These results show that the EMG activity should be considered when we want to know which exercises generate a lower or higher level of activation, which is undoubtedly a criterion for plyometric intensity.

It has also been recommended (Fowler and Lees, 1998) that GRF and momentum be considered factors influencing plyometric intensity, as it allows comparison of different exercise variants. Furthermore, it has been reported that there are no differences in these indicators at the neural level when unilateral and bilateral exercises are performed (Jarvis et al., 2016). However, some level of detail may be lost if only impulse is used to measure intensity, because the maximum level of mechanical stress experienced by the athlete may be consciously hidden (Jarvis et al., 2016). Furthermore, other research corroborates differences between the momentum of different plyometric exercises (Ebben et al., 2010). Therefore, it is recommended to perform a combination between the GRF and the EMG as evidenced in different studies (Van Lieshout et al., 2014; Kossow and Ebben, 2018b). Another important element that should be considered during DJ for reactive purposes is that the mechanical impulse is equal to the product of force times. Thus, the amount of force may be high, but if the amount of time is considerably increased, the reactive strength may be affected. Therefore, the impulse may not be a recommendable metric for analyzing reactive jumps. These means of intensity evaluation are helpful and are constantly evolving, yet they are not part of the practical reality of the sports coach domain. The dynamics of the implementation of plyometric programs do not allow these evaluations to become part of a daily training routine. The reduced rest intervals between exercises, large workgroups, and the unmet need for full-time specialized professionals during training, limit the presence and rigor of valid protocols.

In line with the previous statement, some researchers have proposed to assess training intensity based on contact time (Walsh et al., 2004; Ball et al., 2010), but jump technique (Bobbert et al., 1987a) and individual strategies to minimize GCT may mask the overall intensity values. On the other hand, the height achieved in the jump also has been suggested to assess intensity (Matic et al., 2015b), but this is also influenced by technical and individual strategies (Bobbert et al., 1987a; Bobbert et al., 1987b). Ideally, athletes should maximize JH while minimizing ground contact time. The RSI (Young, 1995) is the proposed metric that solves these problems through the use of different devices that can be easily apply during training (Montoro-Bombú et al., 2022). So far, we have not found in the literature any study assuming that the RSI can be considered a plyometric intensity variable. However, in its assessment, all the characteristics needed for its calculation are present for intensity evaluation. RSI is usually calculated by dividing the jump height by the ground contact time of a DJ (Ebben and Petushek, 2010). Among the studies that evaluated RSI, some implemented a training program (Ramirez-Campillo et al., 2016; Ramirez-Campillo et al., 2018b) and other used a double-blind controlled trial (Bogdanis et al., 2019; Jeffreys et al., 2019; Falch et al., 2022). These studies based their evaluations on traditional jumping tests (DJ and CMJ) and fall heights of ≤40 cm. In one of the studies (Ramirez-Campillo et al., 2019), athletes were required to achieve a contact time of ≤250 m, and failure to do so resulted in retesting. However, the RSI is generally considered highly modifiable, as decreasing contact time compromises jump height as well as the opposite (Newton and Dugan, 2002; Healy et al., 2018). Therefore, to maximize the RSI, the athlete must express an optimal relationship between ground contact time and reactive jump height (Healy et al., 2018). To establish a correct RSI value, it is recommended that researchers and practitioners consider these criteria in future research and carefully familiarize participants with this method prior to the assessment, in order to achieve valid and reliable plyometric intensity scores. Another point to consider is the standardization of the RSI equations. In fact, the values resulting from dividing the JH by the GCT (Young, 1995) are not the same as those resulting from dividing FT by the GCT (Healy et al., 2018); the latter is always higher.

Different research has assessed plyometric intensity using the RPE (Asadi, 2014; Khodaei et al., 2017), and these authors recommend the RPE as a feasible method to assess plyometric intensity. Although using the earlier suggested methods to measure plyometric intensity is more popular among scientists, the necessary instruments are not available to strength and conditioning professionals (Asadi, 2014). For this reason, the research suggests that the RPE provides an innovative and practically compatible alternative for measuring plyometric exercise intensity. One investigation (Asadi, 2014) found a higher RPE for the SJ compared to the DJ35. These results contradict another study showing that the SJ had a lower perceived intensity score than the DJ, while the mean perceived intensity scores of Tuck Jump, Box Jump, the SJ and the DJ were just “moderate” on the RPE scale (Khodaei et al., 2017).

It should be noted that these studies on the RPE take a stand on plyometric intensity without any means of comparison (EMG, RSI, or force platforms). This is of concern, given that previous research (Müller et al., 2020) has found a weak relationship between RPE and reactive jumps power. Therefore, there is a need for further studies assessing the reliability and validity of the RPE for determining plyometric intensity. This is the reason why we did not include the RPE as a method for measuring plyometric intensity in this review. All these criteria help us reinforce the view that plyometric training intensity is a multidimensional factor. Human performance is so diverse that focusing the intensity measurement on a single variable is unwise. It is up to the coach or sport scientist to decide which parameters they want to measure within the training program, and at which stage of the training process it is more convenient to evaluate some parameters rather than others. For example, the DJ followed by a horizontal reactive jump has been shown to have better transfer to the acceleration over short distances, compared to the DJ followed by a vertical reactive jump which has better transfer over longer sprint distances (Meylan et al., 2009; Krejac et al., 2020). Furthermore, there may be no relationship between maximal eccentric GRF production and RSI, because eccentric GRF is favored by increased FH (Peng et al., 2017), while the highest RSI value requires an optimal FH. These two metrics can be assessed for different purposes and at different stages of the training process. Although we do not dismiss the possibility of knowing the maximum GRF during the best RSI performance, these values represent two different criteria for the evaluation of plyometric intensity.

### Considerations on training volume in the included studies

Volume is a component of the training load that must be correctly established. Previous authors conceptualize volume as the total amount of activity to be performed, and it can be constituted by the sum of the work in sessions, weeks, months and years (Bompa and Buzzichelli, 2019). In the case of plyometric training, volume is also a primary criterion. It must be correctly related to the objectives to be achieved, stage of the training process, individual characteristics of the athletes and the metrics related to intensity.

Regarding training volume, a meta-analysis (de Villarreal et al., 2009) recommends a duration of 10 weeks for plyometric training programs and reports that interventions with more than 20 work sessions and more than 50 jumps per session delivered the best results. In the same line of research (Sáez de Villarreal et al., 2012) also indicated that excellent results could be observed in less than 10 weeks (6–8 weeks), reporting that 18 work sessions and more than 80 jumps per session seem to deliver positive adaptations. Even though multiple researchers (Gehri et al., 1998; Luebbers et al., 2003; Markovic et al., 2007; de Villarreal et al., 2008; Chelly et al., 2010; Ramírez-Campillo et al., 2013; Chelly et al., 2014; Ramírez-Campillo et al., 2014; Terzis et al., 2016; Ramirez-Campillo et al., 2018d; Bianchi et al., 2019; Bogdanis et al., 2019; Jeffreys et al., 2019; di Cagno et al., 2020; Ahmadi et al., 2021; do Carmo et al., 2021; Sanchez-Sixto et al., 2021; Falch et al., 2022; Lum et al., 2022) share these positions, we consider training volume an eminently individual parameter. The previously mentioned studies (Table 5) only report group averages in their results, but there is the need to conduct more studies analyzing the individual volume response. The principle of training individualization (Bompa and Buzzichelli, 2019) supports this position. Predetermined volumes may favor some athletes by providing them with an adequate stimulus, but may also be detrimental to others by giving an insufficient or excessive stimulus. We should not overlook the fact that the above mentioned studies based their recommendations on the basis of different measured metrics. In this sense, there might be a difference in training volume to improve the GRF *versus* to improve the RSI. However, this criterion requires further investigation.

Another characteristic commonly observed in the intervention programs was a linear increase of the workload over time. This is widely suggested in the theory of sports training (Bompa and Buzzichelli, 2019), and also responds to the principle of progressive overload (Kasper, 2019), which, in the case of plyometric training means an increase in the tension exerted (Ramirez-Campillo et al., 2020c). Nevertheless, it could be considered a methodological error to establish weekly (Ahmadi et al., 2021) or fortnightly increments (Ramirez-Campillo et al., 2018d; Ahmadi et al., 2021; do Carmo et al., 2021) without prior assessments that would guarantee the sought after adaptations at individualized heights and work volumes. None of the plyometric programs included justifies the rationale for the timing and magnitude of the increase of the load. This review assumes that the gradual increase of the load responds to a criterion of adaptation and control, thus we only recommend increasing the load when each individual athlete shows a positive adaptive response. All the studies included in this systematic review (Table 5) selected jumps volume in an arbitrary and standardized way for all participants. This is undoubtedly a point that needs to be reconsidered in all future research.

All the criteria previously discussed, together with suggestions and conclusions of previous studies (Gehri et al., 1998; Luebbers et al., 2003; Markovic et al., 2007; de Villarreal et al., 2008; de Villarreal et al., 2009; Sedano et al., 2009; Chelly et al., 2010; Khlifa et al., 2010; Sáez-Sáez de Villarreal et al., 2010; Ramírez-Campillo et al., 2013; Chelly et al., 2014; Ramírez-Campillo et al., 2014; Terzis et al., 2016; Eagles et al., 2018; Ramirez-Campillo et al., 2018d; Bianchi et al., 2019; Bogdanis et al., 2019; Jeffreys et al., 2019; Silva et al., 2019; di Cagno et al., 2020; Laurent et al., 2020; Ramirez-Campillo et al., 2020c; Ahmadi et al., 2021; do Carmo et al., 2021; Ramirez-Campillo et al., 2021; Sanchez-Sixto et al., 2021; Falch et al., 2022; Lum et al., 2022), allow us to list the following methodological recommendations that may be useful for the organization of new plyometric training programs for coaches and researchers.

## Conclusion

Several problems regarding the methodological organization and procedure of the proposed plyometric training programs were identified, such as the lack of consensus on a training intensity criterion, a lack of justification for the distribution and orientation of the training volume, as well as a lack of criteria for the individualization of the training process. For these reasons, we established criteria which we consider fundamental for prescribing plyometric training programs. A new criterion for the identification of the subjects’ experience level was established, as well as methodological recommendations for the selection of training intensity and training volume. It is assumed that the drop height should not be understood as the sole determinant of the intensity of plyometric training but should be an integrated factor to determine intensity. These results could be helpful for sport scientists and sport coaches who intend to improve and implement new plyometric training programs. It is also recommended that researchers considered the above recommendations when conduct research procedures.

## Scientific-methodological considerations for the implementation of new plyometric and ballistic training programs

1. Soft surfaces, being more associated with the production of maximal dynamic strength, can be used for the drop jump, when a greater emphasis on countermovement and jump height is sought and when short contact times are not required (DJs with countermovement or Depth Jumps).
2. Soft surfaces can be used for rehabilitation, during the plyometric preparation phase or general preparation phase of athletes, as the training in those phases does not require short ground contact times.
3. Soft surfaces can be more adequate during the first developmental phase of sprint acceleration, where more work on high-force production at relatively low speeds is sought, as well as for quintuple and deca-jumps in general preparation. In addition, they can be used with a wide variety of jumps which constitute the general preparation of top athletes.
4. During training on soft surfaces, athletes should be guided to eliminate heel strikes against the ground so that these are not transferred to hard surface work.
5. Hard surfaces are recommended to be introduced after working on soft surfaces.
6. Hard surfaces are associated with short ground contact times, and bouncing jumps with open knee joint angles.
7. It is recommended not to instruct the landing as “straight leg”. Substitute that instruction with “bouncing fast off the ground with the smallest possible knee angle” or a similar orientation.
8. Hard surfaces are more associated with the development of power, muscle stiffness and reactive strength, so they should be introduced gradually in specific preparation, and kept being used in the pre-competitive and competitive phases.
9. A wide variety of jumps that require short contact times and are primarily based on fast bouncing, can be introduced while training on hard surfaces, as long as metrics such as maximum power output, best reactive strength, etc., are being monitored.
10. Plyometric exercises with less rapid contact times, involving a countermovement and higher drop heights, aiming at producing maximal dynamic strength, increases in ground reaction forces or maximal eccentric force production, can be combined with general or maximal strength work. In this case, Depth Landings, Depth Jumps, Horizontal Drop Jumps, and jumping between high hurdles are recommended.
11. Plyometric exercises with more open knee and hip angles, fast contact times and optimal drop heights, used for the development of maximum power output production, RFD, and Reactive Strength, can be combined with power-oriented weight training work. In this case, exercises such as Tuck Jumps, Drop Jumps, and jumping between medium hurdles are recommended.
12. The combination of fast and slow plyometric exercises (rebounding and countermovement) can be introduced gradually in the stages of transition from general to special preparation or have a specific application during the preparation. For example, if plyometric jumps are used to reinforce maximum dynamic strength during special preparation, or the competitive or tapering phases, Drop Jumps can be combined with horizontal Drop Jumps or Depth Jumps.
13. To determine the level of experience of the participants in a plyometric training program, we recommend the use of the following equation:

$$Level\;of\;plyometric\;experience=\left[\left(A+B+C/2\right)\;/3\right]\;x\;\left(D+E\right)$$

14. The training intensity can be individualized by determining (evaluating) the optimal height for the highest power output, reactive strength, RFD, ground reaction forces, stiffness, reactive jump height, or other performance parameters.
15. Training programs for large groups of athletes should contain different drop heights so that each athlete can train according to his or her level of adaptation. In addition, this facilitates work organization as the group is distributed over several plyo boxes.
16. Training volume can be individualized using two strategies: a) monitoring the individual session so that each athlete does not increase ground contact times, or lose power output or reactive strength, while seeking to maintain the best values for a given metric. Alternatively, b) by establishing a percentage of performance loss compared to the best result in each metric. This ensures that the athlete can train with different performance orientations (as required by the coach), at the same time, it allows athletes to work according to their daily capabilities without having a pre-established number of sets or repetitions that can sometimes be excessive or inadequate.
17. The selection of the optimal drop height should be determined via a specific performance metric. For instance, plyometric training programs aiming at the improvement of maximum power production should establish the DJ fall height that maximize that metric.
18. For a correct execution of the drop, it is recommended that the athlete stands at the edge of the box (not in the center), determines a starting leg, directs the toe up and lets the body fall freely (without pushing forward).
19. We recommend that both the drop height and the training volume (sets and repetitions) should only be increased when real adaptations to the proposed training variables are observed.
