# Supplementary material for: Methodological considerations for determining the volume and intensity of drop jump training. A systematic, critical and prepositive review
Source: Front Physiol. 2023 Apr 21;14:1181781. doi: 10.3389/fphys.2023.1181781 (PMC10160442; doi:10.3389/fphys.2023.1181781)
Supplement: Supplementary file 1 [file Table1.docx]

Supplementary Material 1

**Methodological considerations for determining the volume and intensity of drop jump training. a systematic, critical and prepositive review.**

**Raynier Montoro-Bombú ^1^*, Hugo Sarmento ^1,2^, Carlos Buzzichelli ^3^, Nelio Alfaro Moura^4^, Juan José Gonzales Badillo^5^, Amândio Santos ^1^ and Luís Rama ^1,2^.**

*** Correspondence: Raynier Montoro-Bombú**: [rayniermb@gmail.com](mailto:rayniermb@gmail.com)

**Practical differences between drop jump, depth jump, and depth drop.**

|  | **Drop Jump** | **Depth Jump** | **Depth Drop** |
| --- | --- | --- | --- |
|  |  |  |  |
| Fall height | 20-75 cm | 40 cm – 1.0 m | 0.9 -130 cm |
| RSI characteristics | Shorter ground contact time causes a  higher RSI value. | Longer ground contact time causes a  lower RSI value, despite the higher reactive jump | No RSI |
| Knee joint angle | 130º and 170º | 90º and 135º | 90º and 135º |
| Exercise performance targets | Minimum ground contact time and maximum reactive jump | Maximum height of the reactive jump | Fast termination of the eccentric phase |
| Countermovement range | Minimum | Maximum | Absent |
| Training adaptations | Decreased coupling times and increased reactive strength; increased concentric power output from specific angles. | Increased concentric power output as a result of increased maximum dynamic strength, increased eccentric strength. | Maximum eccentric and isometric strength. |
